# Constitutive expression of *OsDof4*, encoding a C_2_-C_2_ zinc finger transcription factor, confesses its distinct flowering effects under long- and short-day photoperiods in rice (*Oryza sativa* L.)

**DOI:** 10.1186/s12870-017-1109-0

**Published:** 2017-10-19

**Authors:** Qi Wu, Xue Liu, Dedong Yin, Hua Yuan, Qi Xie, Xianfeng Zhao, Xiaobing Li, Lihuang Zhu, Shigui Li, Dayong Li

**Affiliations:** 1 State Key Laboratory of Plant Genomics and National Center for Plant Gene Research, Institute of Genetics and Developmental Biology, Chinese Academy of Sciences, No.1 West Beichen Road, Chaoyang District, Beijing, 100101 China; 2 Rice Research Institute, Sichuan Agricultural University, No. 211 Huimin Road, Chengdu, 611130 China; 3 National Research and Development Center for Coarse Cereal Processing, College of Pharmacy and Biological Engineering, Chengdu University, Chengdu, 610106 China; 4 Key Laboratory of Genome Sciences and Information, Beijing Institute of Genomics, Chinese Academy of Sciences, Beijing, 100101 China; 5 Institute of Turfgrass Science, College of Forestry, Beijing Forestry University, Beijing, 100083 China

**Keywords:** *OsDof4*, Dof transcription factor, Photoperiod, Flowering, Rice (*Oryza sativa* L.)

## Abstract

**Background:**

Dof (DNA binding with one finger) proteins, a class of plant-specific transcription factors which contain a conserved C2-C2-type zinc finger domain, are involved in many fundamental processes. In the Arabidopsis photoperiod response pathway, CDF (CYCLING DOF FACTOR) proteins have a primary role as acting via transcriptional repression of the direct *FLOWERING LOCUS T* (*FT*) activator *CONSTANS* (*CO*). Our previous study indicated that one of *CDF* homologs, *OsDOf12*, was involved in photoperiodic flowering. However, the functional characterization of other rice *CDF* like genes is still in progress. Here, we characterized the function of *OsDof4* in rice.

**Results:**

Phylogenic analysis indicated that OsDof4 is closely clustered into the same subgroup with CDFs and OsDof12. The subcellular localization experiment and transcriptional activity assay suggested that OsDof4 may function as a transcription factor. The diurnal expression pattern indicated that *OsDof4* was regulated by endogenous circadian clock. Overexpression of *OsDof4* led to earlier flowering under natural long-day field conditions (NLDs) and late flowering under natural short-day field conditions (NSDs), respectively. We compared the expression level of key floral genes in vector line and *OsDof4*-ox lines grown under long-day conditions (LDs) and short-day conditions (SDs). Real-time q-PCR results demonstrated that under LDs, *Hd3a*, *RFT1* and *Ehd1* were up-regulated whereas under SDs they were down-regulated. *Hd1* was down-regulated at dusk period independent of photoperiods.

**Conclusions:**

Taken these results together, we may speculate that the abnormal flowering responses in *OsDof4*-ox plants under LDs and SDs might be mediated by *Ehd1* and *Hd1*.

**Electronic supplementary material:**

The online version of this article (10.1186/s12870-017-1109-0) contains supplementary material, which is available to authorized users.

## Background

Plants experience floral transition at the most favorable time to maximize their reproductive success [1–3]. Environmental cues are aligned with endogenous signals to determine the timing of flowering, of which photoperiod has largely been proven to be a principal determinant [4, 5]. Extensive studies have revealed *Arabidopsis thaliana*, a facultative long-day plant (LDP), and rice (*Oryza sativa*), a facultative short-day plant (SDP), share a conserved photoperiodic flowering signaling cascade: *GIGANTEA* (*GI*)-*CONSTANS* (*CO*)-*FLOWERING LOCUS T* (*FT*) [6, 7]. In Arabidopsis, under inductive long-day conditions (LDs) GI and FLAVIN-BINDING, KELCH REPEAT, F-Box 1 (FKF1) form more stable complexes to increase the *CO* expression level, a direct activator of *FT*, by post-transcriptionally degrading CYCLING DOF FACTOR1 (CDF1) which is a *CO* transcriptional repressor, thus promoting flowering [8, 9]. In rice, under inductive short-day conditions (SDs) *OsGI* activates *Heading date 1* (*Hd1*), the counterpart of *CO*, to induce the homolog of *FT*-*Heading date 3a* (*Hd3a*), causing early flowering [10–12]. Nevertheless, there indeed exist some distinct floral molecular mechanisms between these two species [6, 7, 13]. For instance, when switched from inductive SDs to non-inductive LDs, Hd1 converts into a repressor of *Hd3a* by the modification of red-light photoreceptor Phytochrome B (PhyB) [14].

Moreover, compared with Arabidopsis, rice has evolved another unique *Grain number, plant height and heading date 7* (*Ghd7*)*-Early heading date 1*(*Ehd1*)*-Hd3a/Rice FT-like 1* (*RFT1*) flowering procedure because *Ghd7* and *Ehd1* are rice specific genes [6, 7]. *Ehd1* acts as a positive regulator of *Hd3a* and *RFT1* under both SDs and LDs [15]. *Ghd7* is induced by red light and acts as a repressor of *Ehd1* [16, 17]. Therefore, under LDs, *Ghd7* accumulated more and subsequently down-regulates *Ehd1* [17]. In comparison, under SDs, less-induced *Ghd7* attenuates the repression on *Ehd1*, thus activating *Hd3a* and *RFT1* [17]. Furthermore, *Early heading date 2* (*Ehd2*)/*Rice Indeterminate 1* (*RID1*)/*Oryza sativa Indeterminate 1* (*OsId1*), *Early heading date 3* (*Ehd3*), and *Early heading date 4* (*Ehd4*) function as a positive regulator upstream of *Ehd1* under both SDs and LDs [18–22]. On the other hand, *OsMADS51* promotes *Ehd1* under SDs, whereas *OsMADS50* and *OsMADS56* act antagonistically via *OsLFL1-Ehd1* to control flowering under LDs [23, 24]. *Days to heading 8* (*DTH8*) and *Early flowering 1* (*EL1*)/*Heading date 16* (*Hd16*) are inhibitors of *Ehd1* and *Hd3a* under LDs [25–27]. Hence, those regulators upstream of *Ehd1* and *Ghd7* constitute novel flowering network in rice.

Dof (DNA binding with one finger) proteins, a class of plant-specific transcription factors which contain a conserved C_2_C_2_-type zinc finger domain, are involved in many fundamental processes, such as phytohormone response, seed germination, and photoperiodic flowering [28, 29]. In Arabidopsis, systematic and genetic study revealed 4 *Dof*s (including *CDF1*, *CDF2*, *CDF3*, and *CDF5*) acted redundantly to delay flowering by depressing the transcription of *CO* [30]. In rice, 2 *Dof*s, including *rice Dof daily fluctuations 1* (*RDD1*) and *OsDof12*, were previously reported to participate in rice flowering time regulation [31, 32]. *RDD1*-RNAi plants flowered later than wild type [31]. Overexpression of *OsDof12* leads to earlier flowering under LDs by up-regulating *Hd3a* [32].

In present study, we characterized the function of another *Dof* member-*OsDof4* in rice by ectopic expression approaches. Phylogenic analysis showed that protein OsDof4 belonged to the same clade with proteins OsDof12 and CDF1–5. The subcellular localization and transcriptional activity analysis indicated OsDof4 is a plausible transcription factor. Tissue specific profiling showed *OsDof4* is enriched in developing leaves and young panicles, and the diurnal expression rhythm indicated *OsDof4* might be regulated by endogenous circadian clock. Constitutive expression of *OsDof4* gave rise to earlier flowering but delayed flowering, under LDs and SDs, respectively. Further analysis on flowering genes expression levels suggested overexpression of *OsDof4* might affect the flowering time by a regulating *Hd3a* and *RFT1* via *Ehd1* and *Hd1*.

## Methods

### Plant materials and growth conditions

Rice (*Oryza sativa* L.) subspecies *japonica* cultivar Nipponbare was used in this study. The seeds of wild type Nipponbare and the derived transgenic rice lines are maintained in our lab. Rice seeds were sterilized by 3% NaClO solutions, and immersed in water for 2 days, then the germinated seeds were moved onto the soil seed bed in greenhouse with 14 h light supplied. After growth for 28 days, the young seedlings were transplanted to the paddy field. For evaluation of the performance under natural-day conditions, plants were grown in the Experimental Stations (Beijing and Hainan Island) of the Institute of Genetics and Developmental Biology, Chinese Academy of Sciences, China (Beijing: 40°10′N, 116°42′E; Hainan Island: 18°32′N, 110°02′E). The average day lengths in Beijing were approximately 14.3 h at germination, 15 h at 30 days after germination (DAG), 14.8 h at 60 DAG, 13.7 h at 90 DAG. The average day lengths in Hainan Island were approximately 11 h at germination, 11.1 h at 30 DAG, 11.3 h at 60 DAG, 11.7 h at 90 DAG.

Various WT tissues were collected at heading stage to determine the *OsDof4* mRNA levels. For flowering genes expression analysis, the germinated seeds were grown in the growth chamber under SD conditions (10 h light, 28 °C/14 h dark, 24 °C) and LD conditions (14 h light, 28 °C/10 h dark, 24 °C), with the humidity set at approximately 70%. The sampling happened for SD and LD respectively at 28 DAG and 35 DAG. Leaf blades were collected every 3 h under both conditions and stored in liquid nitrogen before total RNA extraction.

### Protein sequence alignment and phylogenic analysis

All the rice Dof protein sequences were downloaded from Rice Genome Annotation Project (RGAP) (http://rice.plantbiology.msu.edu/); the protein sequences of 5 AtDofs were downloaded from The Arabidopsis Information Resource (TAIR) (http://www.arabidopsis.org/). ClustalW program with default parameters was used for protein sequence alignment [33]. The phylogenic tree was constructed using MEGA v5 software by the neighbor-joining method with a bootstrap value of 1000 [34].

### Subcellular localization and rice transformation

The coding sequence without a termination code of *OsDof4* was amplified by PCR using full length-cDNA(FL-cDNA) clone (AK066984) as the template. The FL-cDNA clone was kindly provided by the National Institute of Agrobiological Sciences, Japan (NIAS DNA BANK; http://www.dna.affrc.go.jp/). The PCR products was digested by *Eco*R I and *Bam*H I, and inserted into the binary vector pEZR(K)-LN to create the overexpression vector of *OsDof4* driven by a CaMV *35S* promoter with GFP tagged at the C-terminus of OsDof4. The primers used were list in Additional file 1: Table S1. To construct OsDof4 overexpression lines, the overexpression vector (p*35S*::*OsDof4*:*GFP*) and control vector (p*35S*::*GFP*) were transformed to *Agrobacterium tumefaciens* strain LBA4404 (Shanghai Weidi Biotechnology, China) by electroporation, and introduced into wild type cultivator Nipponbare following the protocol as described previously [35].

For subcellular localization of OsDof4, the empty vector p*35S*::*GFP* or p*35S*::*OsDof4*:*GFP* with nuclear marker construct pSAT6-mCherry-VirD2NLS [36] were transiently co-expressed in rice protoplasts by means of PEG–mediated transformation [37]. The fluorescent signals were observed by a fluorescence confocal microscope (Zeiss Axio Imager.Z2, http://www.zeiss.com).

### Transcriptional activation assay

The transcriptional activity of *OsDof4* was evaluated by Dual-Luciferase Reporter Assay System (Promega, http://www.promega.com). The plasmids used in this assay were kindly provided by Prof. Shouyi Chen (Institute of Genetics and Biology, Chinese Academy of Science). pGAL4-LUC in which a minimal *35S* plus five copies of GAL4 binding elements driving the firefly luciferase coding sequence was used as reporter. pTRL with *AtUbiquitin3* promoter driving the *Renilla reniformis* luciferase gene was used as internal control [38]. The full-length coding sequence of *OsDof4* was amplified with the primers (Additional file 1: Table S1) by PCR, and recombinated using NEBuilder® HiFi DNA Assembly Master Mix Kit (NEB, http://www.neb.com) into the sites *Bam* HI and *Sal* I at the 3′ end of GAL4 DNA binding domain coding sequence in pGAL4DBD to generate the effector plasmid pGAL4DBD-OsDof4. pGAL4DBD and pGAL4DBD-VP16 were used respectively as the negative and positive effector [38].

The plasmids with the content ratio of effector: reporter: internal control plasmids at 6:6:1(6μg:6μg:1μg) were co-transfected into rice leaf protoplasts via PEG mediated treatment [37]. After incubation in dark at 28 °C for 16 h, the relative luciferase activity(represented by the ratio of *Firefly* LUC activity to *Renilla* LUC activity) was measured by the GloMax 20–20 luminometer based on a Dual-Luciferase Reporter Assay System [38].

### RNA extraction and real-time PCR analysis

The Total RNA was isolated from different rice tissues using Trizol reagent (Invitrogen, California, USA) according to the manufacturer’s instruction. DNA digestion was done by DnaseI (Takara, Japan), and first-strand cDNA was obtained by GoScript Reverse Transcription System Kit (Promega, http://www.promega.com). Real-time PCR was performed using EvaGreen qPCR Master Mix (Abm, Canada) on a real-time PCR System (Bio-Rad CFX96) with the specific primers. The real-time PCR program consists of 95 °C for 3 min and 42 cycles of 95 °C for 5 s, 60 °C for 15 s. Three replicates were performed for each gene and for each analysis two biological repeats were done. All the specific primers used were listed in Additional file 1: Table S1.

### Statistical analysis

The data of transcriptional activity assay in Fig. 3b and the agronomic traits in Fig. 5c-f was subjected to Student’s *t*-test analysis using Microsoft Excel 2015.

## Results

### *OsDof4* encodes a Dof transcription factor

**Fig. 1:**
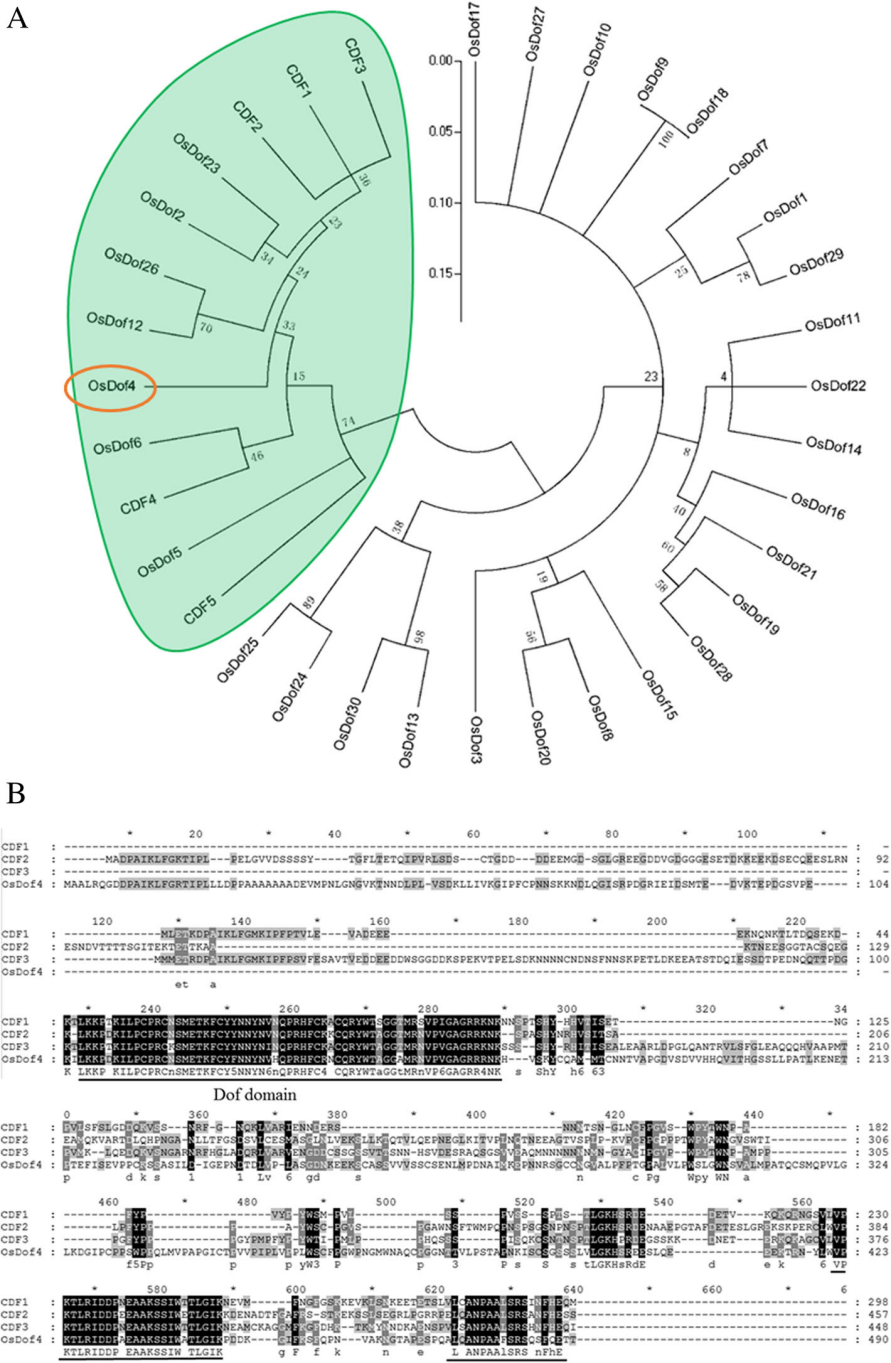
Phylogenic analysis and protein structure of OsDof4 homologs. **a** Phylogenic relationship between OsDof4 homologs from rice and Arabidopsis. An unrooted neighbor-joining tree was reconstructed based on the full–length amino acid sequences of rice Dofs and CDFs from Arabidopsis. Numbers stands for the bootsrap values along the branches. The protein sequences of rice Dofs were downloaded from RGAP, and the protein sequences of 5 Arabidopsis Dofs were downloaded from TAIR. **b** Protein sequence comparasion between OsDof4 and CDF1–3. The identical residues are highlighted in black,and the similar ones are in gray. The conserved Dof domain and two putative motifs were underlined

We are quite interested in rice *Dof* genes because of their importance on various biological processes. In Arabidopsis, CDFs play key roles in photoperiodic flowering time [30, 39]. To explore the relationship between CDFs and rice Dof homologs, we conducted phylogenetic analysis with a neighbor-joining method. The phylogenic tree indicated that OsDof2, OsDof4, OsDof5, OsDof6, OsDof12, OsDof23 and OsDof26 are closely clustered with CDFs (Fig. 1a), thus we named them OsCDFs (*Oryza sativa* CDFs). In our previous study, we characterized the function of *OsDof12* and found that, overexpression of *OsDof12* led to earlier flowering [32]. To further investigate the biological roles of *CDF*s, we obtained *OsDof4* overexpression lines. We screened and found the transgenic lines harboring *OsDof4* (Locus Number: *LOC_Os01g17000*) coding sequence (CDS) showed abnormal flowering responses. According to the Rice Genome Annotation Project database (http://rice.plantbiology.msu.edu/), the reading frame of *LOC_01g17000* totals 1473 base pairs (bp) with an intron of 2140 bp, encoding a protein of 490 amino acids. By searching the Pfam database(http://pfam.xfam.org), we found that the deduced protein is characteristic of DNA binding with one finger (Dof) domain (PF02701) located at N-terminus from 112 to 168 amino acids. Besides, the protein sequences aligned with CDF1, CDF2 and CDF3 indicated that OsDof4 shares highly conserved Dof domain and two motifs at the C-terminus with CDFs (Fig. 1b).

### OsDof4 is localized in nucleus

**Fig. 2:**
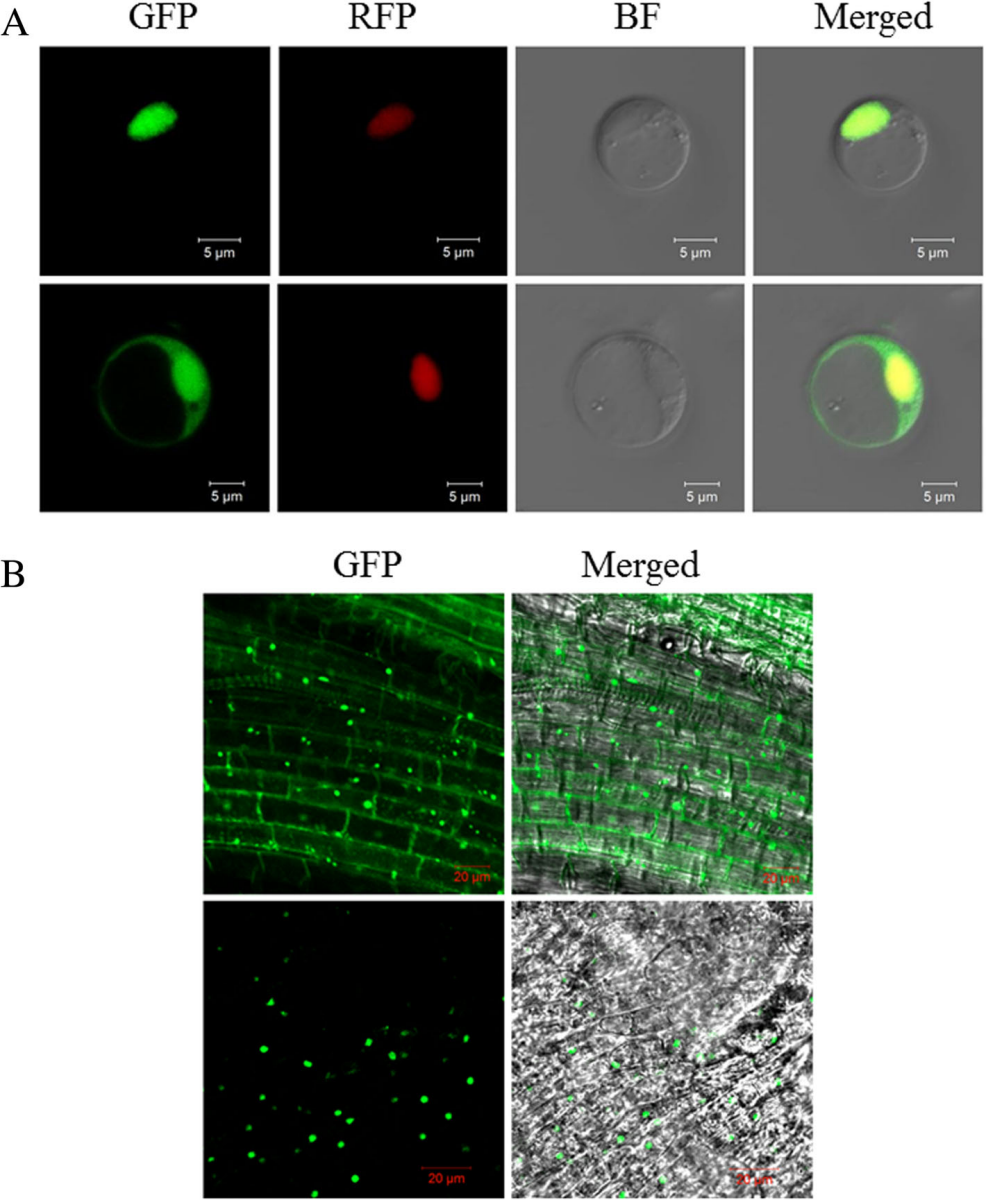
Subcellular localization of OsDof4. **a** Subcellular localization of OsDof4 in rice protoplasts. The vector *35S::OsDof4-GFP* or *35S::GFP* with the nuclear marker plasmids pSAT6-mCherry-VirD2NLS were co-transformed into and transiently expressed in rice protoplasts. Left to right: GFP fluorescence signal, RFP fluorescence signal, Bright-field signal, and Merged signal. Top panel: localization of OsDof4-GFP fusion proteins. Bottom panel: localization of free GFP proteins. Scale bar = 5um. The excitation wavelengths for GFP and RFP detection were respectively 488 nm and 587 nm. **b** Microscopic observation of the fresh root tips in 40-day-old transgenic plants. The top panel indicated the images acquired from *35S::GFP* plants; The bottom panel indicated the images acquired from *35S::OsDof4-GFP* plants. Left: GFP fluorescence signal; Right: Merged signal. Bar = 20um

In higher plants, several cases have shown Dof proteins by binding to promotor region regulate the expression of downstream targets in nucleus [40]. To gain more insight into the function of OsDof4, the full coding region of OsDof4 was fused with GFP, and introduced into rice leaf protoplasts, where the genes were transiently expressed under the control of *CaMV 35S* promotor. The fluorescent signal of OsDof4-GFP was specifically detected in nucleus where exclusively co-localized with mCherry-VirD2NLS (a nuclear marker) (Fig. 2a). In comparison, the free GFP signal was distributed in both nucleus and cytoplasm (Fig. 2a). Meanwhile, we analyzed the in vivo localization of OsDof4-GFP in transgenic plant roots. Similar to the results obtained in rice leaf protoplasts, OsDof4-GFP was predominantly distributed in nucleus whereas free GFP equally appeared in nucleus and cytoplasm (Fig. 2b), further confirming OsDof4 is nuclear-localized. Consequently, we proposed that OsDof4 might function as a nuclear transcription factor.

### OsDof4 has the capability of transcriptional activation

**Fig. 3:**
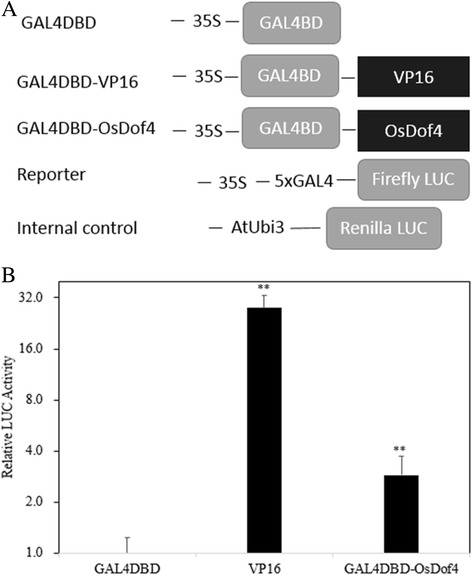
Transcriptional activity assay of *OsDof4*. **a** Schematic representation of effectors, reporter, and internal control plasmids structure. **b** The transcriptional activity of each effector. GAL4DBD and GAL4DBD-VP16 represented respectively negative and positive control effector. The plasmids of effector, reporter, and internal control were co-transformed into rice protoplasts, after overnight incubation in dark at 28 °C, the relative LUC activity of each effector was measured. With the relative LUC activity of GAL4DBD normalized to 1, obviously GAL4DBD-VP16 and GAL4DBD-OsDof4 showed higher transcriptional activity. Values are means ± SE (*n* = 5). Error bar indicates SE. The double asterisks indicate significant difference (*p* < 0.01) compared to GAL4DBD value according to student’s *t* test

To confirm whether OsDof4 is a transcription factor, we tested the transcriptional activation ability by using a dual-luciferase reporter (DLR) assay system in rice protoplasts. The full coding sequence of OsDof4 was fused in-frame with pGAL4DBD to generate effector construct pGAL4DBD-OsDof4. With pGAL4DBD and pGAL4DBD-VP16 setting respectively as negative and positive control (Fig. 3a), we conducted a transcriptional activity assay. We found that pGAL4DBD-OsDof4 exerted obviously enhanced transcriptional activation ability compared with pGAL4DBD (Fig. 3b), implying that OsDof4 is capable of activating transcription, which further confirmed that *OsDof4* encodes a rice transcription factor.

### Expression of *OsDof4* is ubiquitous and diurnal

**Fig. 4:**
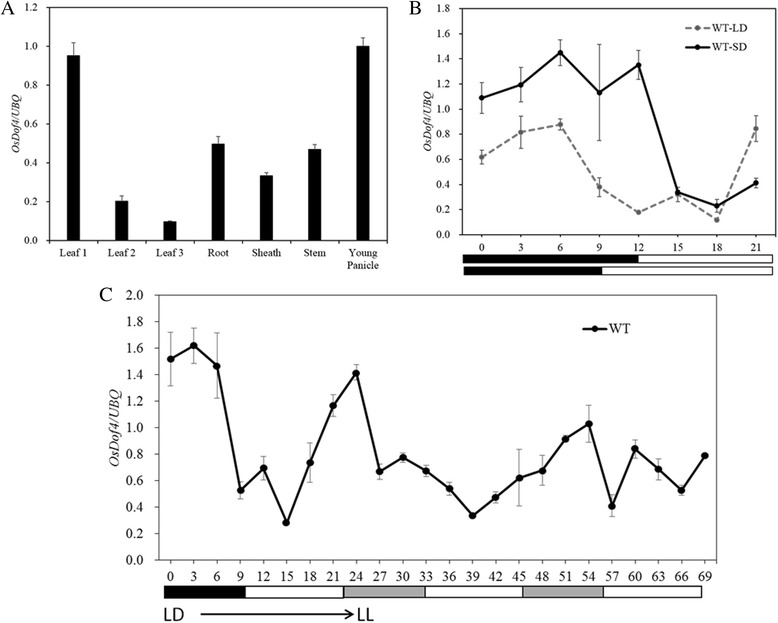
Tissue-specific and diurnal expression profiles of *OsDof4* in WT. **a** *OsDof4* mRNA expression in various rice tissues. All the rice tissues were harvested from WT at heading stage. **b** Diurnal expression pattern of *OsDof4*. Leaf blades from 28- and 35-day-old WT grown under SDs and LDs, respectively, were collected for expression analysis. The solid line standed for the expression values under SDs, and the dotted line standed for the ones under LDs. **c** Free running *OsDof4* expression curve in continuous light conditions. 4-week-old WT were firstly entrained in LDs for 2 weeks and then moved to constant light conditions (LLs). The leaf blades were harvested every three hours for 24 h in LDs and for 48 h in LLs. Values are means ± SE (*n* = 3). Error bar indicates SE

To determine the tissue specific expression profiles of *OsDof4*, we determined the *OsDof4* mRNA abundance in various rice tissues by real-time PCR. *OsDof4* showed constitutive expression pattern, and was most abundantly expressed in developing leaves (leaf 1) and young panicles, followed by root, stem, sheath and developed leaves (leaf 2, leaf 3) (Fig. 4a). *OsDof4* exhibited diurnal expression pattern under both SDs and LDs, the mRNA abundance was much higher at day time than that at night time (Fig. 4b). The diurnal rhythm of *OsDof4* mRNA level indicated that *OsDof4* might be regulated by an endogenous circadian clock. To investigate whether *OsDof4* is driven by circadian clock, we examined the *OsDof4* transcript level in constant light conditions. Four-week-old wild type (WT,cv. Nipponbare) seedlings were firstly entrained in LDs for two weeks and then moved to constant light conditions (LLs). The leaf blades were harvested every three hours for 24 h in LDs and for 48 h in LLs. Then the collected samples were subjected to RNA extraction and real-time PCR analysis. The result showed that, though the amplitude in LLs was attenuated than that in LDs, the phases in both conditions were very alike (Fig. 4c). Thus, we postulated that *OsDof4* is regulated by circadian clock.

### Overexpression of *OsDof4* promotes flowering under NLDs but delays flowering under NSDs

To elucidate the biological function of *OsDof4*, we constructed transgenic lines in which *OsDof4* was driven under the control of *CaMV 35S* promotor (35S::*OsDof4*), and the empty vector lines were set as control. In total, we obtained eighteen and four positive lines harboring 35S::*OsDof4* and empty vector, respectively. We randomly chose eight 35S::*OsDof4* (including line *OsDof4*-ox-1/2/4/6/9/10/13/18) lines and one empty vector line to analyze the expression level of *OsDof4* by using real-time PCR, and found all *OsDof4*-ox lines showed obviously much higher *OsDof4* mRNA level than that in WT or empty vector plants (Additional file 2: Figure S1).

**Fig. 5:**
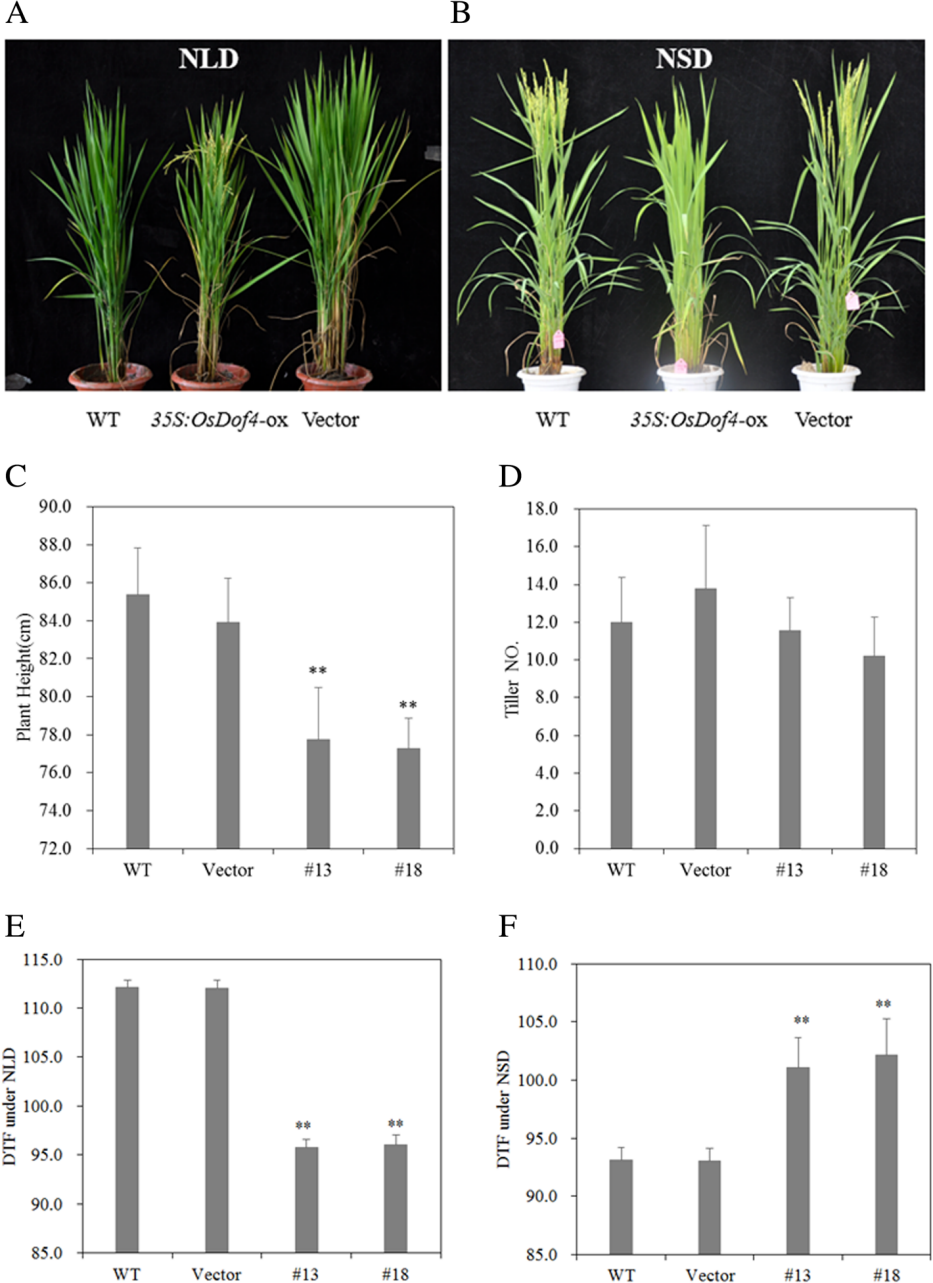
Agronomic traits comparison between *OsDof4*-ox and control plants. **a**-**b** Morphologic comparision between WT, Vector and *OsDof4*-ox plants at heading stage. NLD and NSD represent for the pictures acquired in Beijing and Hainan Island, respectively. **c**-**d** Statistical data of plant height (**c**) and tiller number (**d**). The plant height and tiller number were measured after mature stage. **e**-**f** Statistical data of flowering time under NLD (**e**) and NSD (**f**). DTF represents for the days from germination to flowering. Values are means ± SE (*n* = 20). Error bar indicates SE. The double asterisks indicate significant difference (p < 0.01) compared to control according to student’s *t* test

To investigate the biological effects that *OsDof4* may bring, we grew the *OsDof4*-ox lines and vector lines in Beijing and Hainan experimental fields, respectively. We observed and statistically analyzed the agronomic traits of two *OsDof4*-ox lines (*OsDof4*-ox-13, *OsDof4*-ox-18), WT, and one vector line. Comparison of morphologic phenotypes between WT, vector plants and *OsDof4*-ox-13, *OsDof4*-ox-18 plants revealed that the plant height of WT or vector plants reached about 84 cm, while *OsDof4*-ox-13, *OsDof4*-ox-18 plants only were 78 cm(cm) in height which were significantly dwarf than WT or vector plants (Fig. 5a-c). Meanwhile, we found the tiller numbers in each lines were comparable (Fig. 5d).

Besides, we found the flowering time in *OsDof4*-ox-13, *OsDof4*-ox-18 plants were significantly different from that in WT or vector plants. When grew in Beijing (40°10′N, 116°42′E), which was more like natural long-day field conditions (NLDs), *OsDof4*-ox-13, *OsDof4*-ox-18 plants promoted flowering by 18 days than WT or vector plants on average (Fig. 5a, e). Interestingly, when grew in Hainan Island (18°32′N, 110°02′E), which was more like natural short-day field conditions (NSDs), *OsDof4*-ox-13, *OsDof4*-ox-18 plants delayed flowering by about 10 days than WT or vector plants (Fig. 5b, f). These results implied constitutive expression of *OsDof4* affected the photoperiodic flowering response under both conditions.

### Flowering genes expression are affected in *OsDof4*-ox plants

To gain more insight into the abnormal flowering responses and elucidate the corresponding molecular mechanisms in *OsDof4*-ox plants, we examined the key flowering regulators expression levels by conducting real-time PCR. *OsDof4*-ox plants and vector plants were grew under controlled conditions. Leaf blades from 28-day-old and 35-day-old seedlings cultivated respectively under SDs and LDs were harvested for expression analysis.

**Fig. 6:**
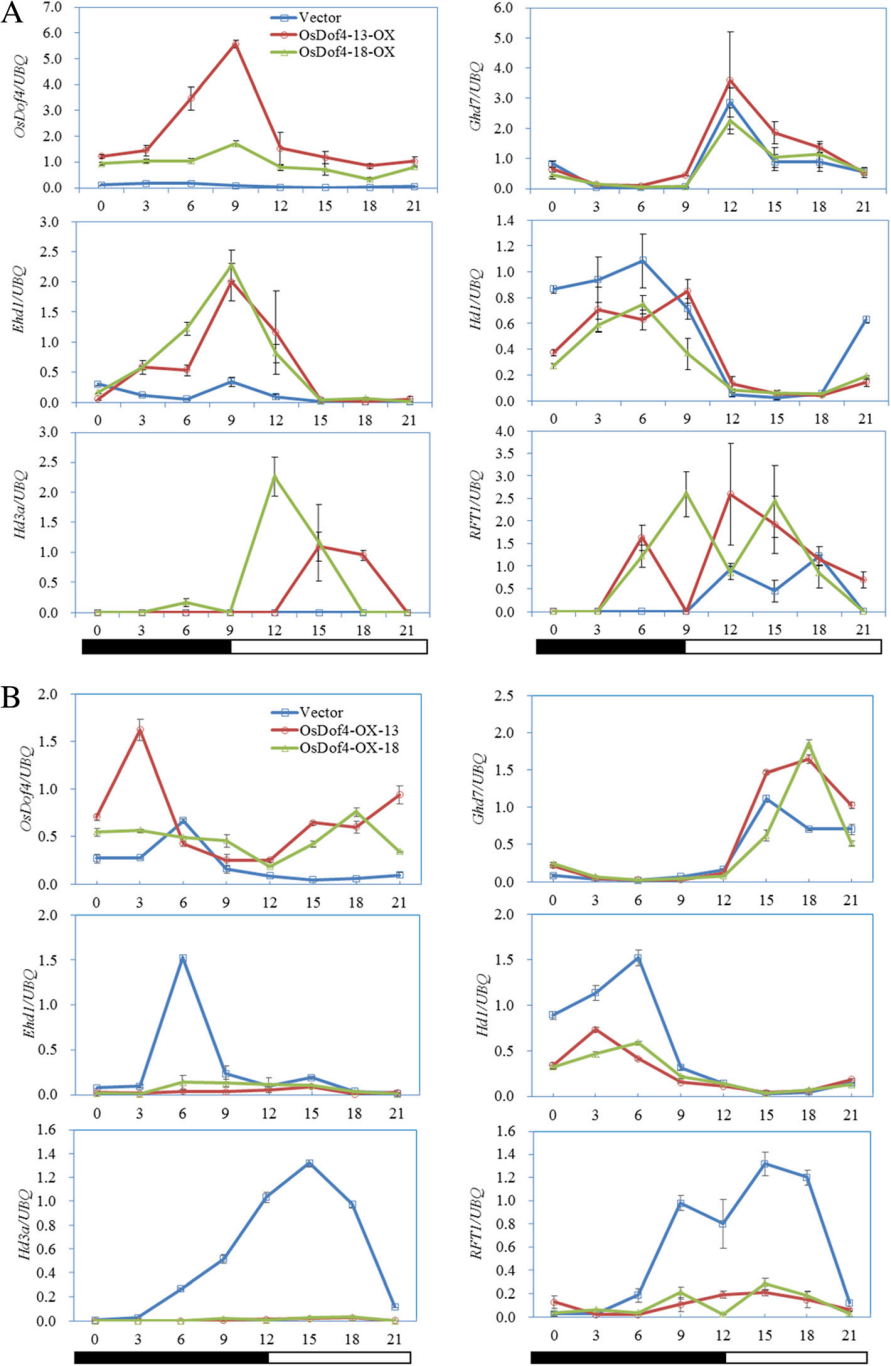
Expression analysis of rice flowering genes in vector and *OsDof4*-ox plants. **a**-**b** mRNA levels of flowering genes under LDs (**a**) and SDs (**b**) were detected by real-time PCR. Leaf blades from 28- and 35-day-old WT grown under SDs and LDs, respectively, were collected for expression analysis. Blue lines represent for the expression curves of flowering genes in vector plants; Red and green lines respectively represent for the ones in *OsDof4*-ox-13/−18 plants. Values are means ± SE (n = 3). Error bar indicates SE

Under long-day conditions (LDs), two florigen genes, *Hd3a* and *RFT1*, were significantly up-regulated in *OsDof4*-ox-13, *OsDof4*-ox-18 plants compared with vector plants (Fig. 6a). Furthermore, we analyzed the expression levels of flowering genes upstream of *Hd3a* and *RFT1*, including *Ghd7*, *Ehd1*, and *Hd1*. The transcription level of *Ghd7* was unimpaired in *OsDof4*-ox-13, *OsDof4*-ox-18 plants (Fig. 6a). *Hd1* in *OsDof4*-ox-13, *OsDof4*-ox-18 plants was down-regulated in the dusk period but was not obviously affected in the light period than that in vector plants (Fig. 6a). Strikingly, the expression level of *Ehd1* in *OsDof4*-ox-13, *OsDof4*-ox-18 plants was obviously much higher than that in vector plants (Fig. 6a).

Under SD conditions (SDs), the transcription levels of *Hd3a* and *RFT1* mRNAs were severely lower in *OsDof4*-ox-13, *OsDof4*-ox-18 plants (Fig. 6b). Similar to the observation under LDs, *Ghd7* mRNA levels were comparable in *OsDof4*-ox plants and vector plants (Fig. 6b), and *Hd1* in *OsDof4*-ox-13, *OsDof4*-ox-18 plants was down-regulated in the dusk period but was not obviously affected in the light period than that in vector plants (Fig. 6b). However, the expression level of *Ehd1* in *OsDof4*-ox-13, *OsDof4*-ox-18 plants was strongly depressed than that in vector plants (Fig. 6b).

Taken together, these results indicated overexpression of *OsDof4* may up-regulate but down-regulate *Hd3a* and *RFT1* under LDs and SDs, respectively, by modulating the expression levels of *Ehd1* and *Hd1*.

## Discussion

In plants, *Dof* genes have been well documented participating in various important developmental events, including flowering [28, 29]. There has much progress in understanding *Dof* genes involved floral mechanisms in model dicot plant-Arabidopsis, however, the counterpart in rice remain rather limited. Our previous study showed overexpression of *OsDof12* promoting flowering by up-regulating *Hd3a* under LDs in rice [32]. In the present study, we demonstrated that constitutive expression of another *Dof* member, *OsDof4* in rice, could lead to distinct flowering phenotypes under LDs and SDs, respectively. Further evidences implied affected expression of *Ehd1* might be responsible for the abnormal flowering time in *OsDof4* overexpression plants. Taken together, our study clearly reveals that *OsDof4* plays a pivotal role in rice flowering pathway.

Tissue specific expression profile displayed that *OsDof4* was ubiquitously expressed in root, stem, leaf and young panicle (Fig. 4a), suggesting *OsDof4* might be a versatile regulator. Indeed, *OsDof4*-ox lines showed significantly reduced plant height than control lines (Fig. 5a-c). Interestingly, our recent study indicated that another *Dof* member in rice-*OsDof12* may be involved in plant height regulation [41]. Considering proteins OsDof4 and OsDof12 are clustered into one clade, we surmised that *OsDof4* may play an analogic role as *OsDof12* does in plant height regulation. However, its underlying molecular mechanisms remain to be further investigated. Endogenous circadian clock is involved in many biological processes by regulating various downstream transcriptional regulators [42, 43]. The five Arabidopsis *Dof* members *CDF1*, *CDF2*, *CDF3*, *CDF4* and *CDF5* all showed circadian clock-regulated rhythms [30, 39], meanwhile, the daily oscillations in *Rdd1* expression also indicated it was circadian-regulated in rice [31]. By observing the expression rhythm of *OsDof4* in LLs, we speculated that *OsDof4* might be also controlled by endogenous circadian clock. Notably, mRNA level of *Rdd1* peaked at subjective light period and, *OsDof12* transcriptional level decreased under dark condition. By contrast, *OsDof4* mRNA transcripts accumulated and peaked at light period but, declined at dark period (Fig. 4b-c). These observations imply that though OsDof4 shares similar protein sequence with Rdd1 and OsDof12, still, there might exist distinguished biological function between them because of the differences in diurnal expression profiles.

In Arabidopsis, Fornara et al. (2009) systematically misexpressed 26 *Dof* members in companion cells by using the *SUCROSE TRANSPORTER 2* (*SUC2*) promoter, and found that 5 *Dof* members overexpressors transgenic lines displayed delayed flowering time under LDs [30]. Phylogenic analysis suggested those 5 *Dof* members, including *CDF2*, *CDF3*, *CDF4*, *CDF5* and *COG1*, clustered in the same clade with *CDF1* which had been reported to delay flowering when overexpressed by CaMV *35S* promoter [30, 39]. Coincidently, our result indicated *OsDof4* also belongs to the same subgroup, suggesting *OsDof4* might share similar conserved role in flowering pathway with those Arabidopsis *Dof* members. We conducted reverse-genetics approaches to characterize the function of *OsDof4* because there were no corresponding mutants in the rice mutant database. Observation of the *OsDof4*-ox lines flowering time come to a conclusion that overexpression of *OsDof4* caused opposite effects on flowering under LDs and SDs, respectively. It is of interest to note that *OsDof12*-ox lines also flowered earlier under LD but no clear difference occurred under SDs [32]. Moreover, *CDF*s overexpressors flowered later under LDs rather than under SDs in Arabidopsis [30, 39]. Therefore, as a homolog of *OsDof12* and *CDF*s, *OsDof4* displayed novel roles in flowering regulation especially under SDs.

To decipher the mechanisms underlying the affected photoperiod sensitivity in *OsDof4*-ox plants, we tested the expression of key floral regulators. Up to now, the largely accumulated evidences have suggested that *Hd3a* and *RFT1* are indispensable for rice flowering [44, 45]. We found that *Hd3a* and *RFT1* transcript-level expression in *OsDof4*-ox were higher but lower than that in control plants under LDs and SDs (Fig. 6a-b), respectively, which were closely corresponding with the flowering time. Considering *Hd1* and *Ehd1* are the main transcriptional regulators upstream of *Hd3a* and *RFT1* [11, 15, 17], we further carried out real-time PCR to detect their expression. The results showed that *Ehd1* was positively regulated in *OsDof4*-ox plants under LDs (Fig. 6a). However, under SDs the scenario was opposed where *Ehd1* was strongly repressed in *OsDof4*-ox plants (Fig. 6b). Meanwhile, in *OsDof4*-ox plants, *Hd1* was not obviously affected in the light period but, repressed at dust period independent of photoperiod (Fig. 6a-b). Previous studies have revealed that, *Ghd7*, *OsGI*, *OsMADS50*, *OsMADS51*, *OsMADS56*, *Ehd2* and *DTH8* are involved in regulation of *Hd1* or *Ehd1* [12, 16–20, 23–25, 46]. Therefore, we also tried to figure out whether the expression variation in *Ehd1* or *Hd1* in *OsDof4*-ox plants were mediated by these regulators under LDs but, found that all these regulators displayed robust expression as control plants did (Additional file 3: Figure S2). Previous reports have revealed that *Ehd1* functions as a floral promotor under both LDs and SDs and, *Hd1* acts as a florigen activator under SDs but converts into a repressor under LDs [10, 11, 14, 15, 45]. Consequently, in combination with our experimental data, we deduced that the impaired transcript-level expression of *Hd3a* and *RFT1* or flowering time in *OsDof4*-ox plants was probably mediated by *Ehd1*, *Hd1* and some other factors.

In rice, a near-isogenic line carrying a weak photoperiod-sensitive allele of *EL1/Hd16* (Koshihikari) was reported to promote flowering under LDs and inhibit flowering under SDs [26]. Meanwhile, *Hd16*-RNAi plants showed earlier flowering under LDs and showed later flowering under SDs than WT plants [26]. The flowering phenotypes in *Hd16*-RNAi lines under SDs and LDs are very similar to that in *OsDof4*-ox lines. Further analysis showed that *Hd16* controls the flowering time via modulating the expression of *Ehd1* and its downstream *Hd3a* and *RFT1*, which indicates *Hd16* and *OsDof4* might share the same pathway. However, from our transcriptome date (not published) we found *Hd16* was not affected under SDs and LDs in *OsDof4*-ox lines, thus we speculated that *OsDof4* may not regulate *Hd16* at transcriptional level. Anyway, the exact genetic relationship between *OsDof4*, *Hd16* and *Ehd1* should be investigated in future work.

Subcellular localization (Fig. 2a-b) and transcriptional activity assay (Fig. 3b) together strongly indicated OsDof4 functions as a transcription factor, but that whether OsDof4 directly regulates *Ehd1* and *Hd1* or not should be further revealed. Besides, more recently, Nemoto et al. [47] suggested that, under LDs Hd1 and Ghd7 form a complex binding to the *cis*-regulatory elements in *Ehd1* and repress it. Therefore, to which extent the expression variation in *Hd1* accounts for the impaired *Ehd1* expression, at least under LDs, remains to be investigated.

Overexpression of *OsDof4* leads to earlier but late flowering under LDs and SDs, respectively. On the contrary, whether the plants harboring down-expressed or knock-outed *OsDof4* induce opposed effects should be further validated. Actually, to fully understand the role of *OsDof4*, we successfully constructed *OsDof4* knockout mutant-*osdof4* in wild type Nipponbare background, by using gene editing tool-CRISPR/Cas9 [48]. Comparison of the *OsDof4* allele sequences between WT and *osdof4*-KO lines revealed that there was a 4-bp deletion in line SG1546–3 and multi-site deletion in another line SG1546–6, resulting in a premature stop codon in the open reading frame (data not shown). Thus, we concluded that *OsDof4* was specifically knocked out in *osdof4*-KO lines. Therefore, these results suggested that loss-of-function in *OsDof4* do not affect the flowering time which may result from the functional redundancy of other *Dof* family genes. Multi-*Dof*-mutation approaches in future study will provide more cues to determine which *Dof* genes compensate the loss-of-function effect of *OsDof4* on flowering time.

## Conclusions

Collectively, we demonstrate that *OsDof4* plays important roles in regulating rice flowering time. Overexpression of *OsDof4* led to earlier flowering under LDs and late flowering under SDs, respectively. Our results imply that the abnormal flowering responses in *OsDof4*-ox plants under LDs and SDs might be mediated by *Ehd1* and *Hd1*. This finding could help us better understand the photoperiodic flowering in rice.

## Additional files

Additional file 1: Table S1. The primers sequences used in this study. (DOCX 19 kb)

Additional file 2: Figure S1. *OsDof4* expression levels in WT, vector line and *OsDof4*-ox lines. Leaf blades from plants before heading stage were collected for real-time PCR. Values are means ± SE (*n* = 3). Error bar indicates SE. (TIFF 1080 kb)

Additional file 3: Figure S2. Expression analysis of key floral genes in vector and *OsDof4*-ox plants under LDs. mRNA levels of flowering genes under LDs were detected by real-time q-PCR. Leaf blades from 35-day-old WT grown under LDs were collected for expression analysis. Blue lines represent for the expression curves of flowering genes in vector plants; Red and green lines respectively represent for the ones in *OsDof4*-ox-13/−18 plants. Values are means ± SE (n = 3). Error bar indicates SE. (TIFF 2564 kb)

## Abbreviations

CDF: CYCLING DOF FACTOR
CO: CONSTANS
Dof: DNA binding with one finger
DTH8: Days to heading 8
Ehd1: Early heading date 1
Ehd2: Early heading date 2
Ehd3: Early heading date 3
Ehd4: Early heading date 4
EL1: Early flowering 1
FKF1: FLAVIN-BINDING KELCH REPEAT, F-Box 1
FT: FLOWERING LOCUS T
Ghd7: Grain number, plant height and heading date 7
GI: GIGANTEA
Hd1: Heading date 1
Hd16: Heading date 16
Hd3a: Heading date 3a
LDs: long-day conditions
NLDs: natural long-day field conditions
NSDs: natural short-day field conditions
OsId1: *Oryza sativa* Indeterminate 1
RDD1: Dof daily fluctuations 1
RFT1: Rice FT-like 1
RID1: Rice Indeterminate 1
SDs: short-day conditions
