# Supplementary material for: Constitutive expression of OsDof4, encoding a C2-C2 zinc finger transcription factor, confesses its distinct flowering effects under long- and short-day photoperiods in rice (Oryza sativa L.)
Source: BMC Plant Biol. 2017 Oct 19;17:166. doi: 10.1186/s12870-017-1109-0 (PMC5649077; doi:10.1186/s12870-017-1109-0)
Supplement: Supplementary file 1 — The primers sequences used in this study. (DOCX 19 kb) [file 12870_2017_1109_MOESM1_ESM.docx]

**Additional file 1: Table S1 The primers sequences used in this study.**

| Construct name | Primer-f (5’-3’) | Primer-r (5’-3’) |
| --- | --- | --- |
| p35S::OsDof4:GFP | GAATTCATGGCCGCGCTTAGGCAG | GGATCCCGAGTCGTCTCTTGAAACGA |
| pGAL4DBD-OsDof4 | GAGCTCTCTAGAACTAGTGGATCCATGGCCGCGCTTAGGC | CCGGGCCCCCCCTCGAGGTCGACTCAAGTCGTCTCTTGAA |
| q-*UBQ* | AACCAGCTGAGGCCCAAGA | ACGATTGATTTAACCAGTCCATGA |
| q-*Ehd1* | CCTACAGTGATTATGGCTTCA | GTGCTGCCAAATGTTGCTC |
| q*-Hd1* | TCAGCAACAGCATATCTTTCTCATCA | TCTGGAATTTGGCATATCTATCACC |
| q*-RFT1* | TGACCTAGATTCAAAGTCTAATCCTT | TGCCGGCCATGTCAAATTAATAAC |
| q*-Hd3a* | GCTCACTATCATCATCCAGCATG | CCTTGCTCAGCTATTTAATTGCATAA |
| q*-Ghd7* | GCTTGAACCCAAACACGG | CTCATCTCGGCATAGGCTT |
| q*-OsDof4* | CCTGTTGTTCCAATCCCTCT | GTTGACGGCAGCACAGTAGT |
| q*-OsGi* | ATTTGGCAAACTTCCTCACC | ACCTTTCTCCAACCCTGATG |
| q*-DTH8* | CAGGAGTGCGTGTCGGAGTT | GGTCGTCGCCGTTGATGGT |
| q*-Ehd2* | AGCGATTTCAATCCATGCAAG | AGCAACTACGACTCACCATTAAC |
| q*-OsMADS50* | CAGGCCAGGAATAAGCTGGAT | TTAGGATGGTTTGGTGTCATTGC |
| q*-OsMADS51* | GTTTGCTCTGCTCCTACTC | ACTCCTCCTCCAGCATTGAA |
| q*-OsMADS56* | GACCGCTATAAAGCATACACA | TCATGTGGTTAGCCACCAGC |
