# Supplementary figures and images for: Constitutive expression of OsDof4, encoding a C2-C2 zinc finger transcription factor, confesses its distinct flowering effects under long- and short-day photoperiods in rice (Oryza sativa L.)
Source: BMC Plant Biol. 2017 Oct 19;17:166. doi: 10.1186/s12870-017-1109-0 (PMC5649077; doi:10.1186/s12870-017-1109-0)

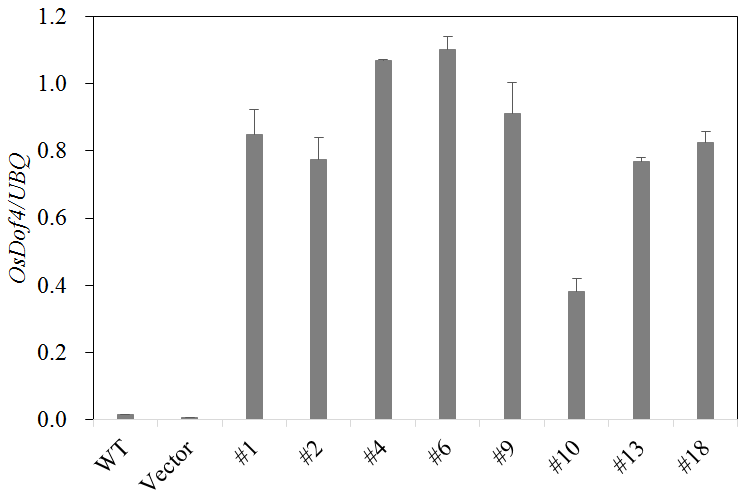

Supplement: Supplementary file 2 — OsDof4 expression levels in WT, vector line and OsDof4-ox lines. Leaf blades from plants before heading stage were collected for real-time PCR. Values are means ± SE (n = 3). Error bar indicates SE. (TIFF 1080 kb) [file 12870_2017_1109_MOESM2_ESM.tif]

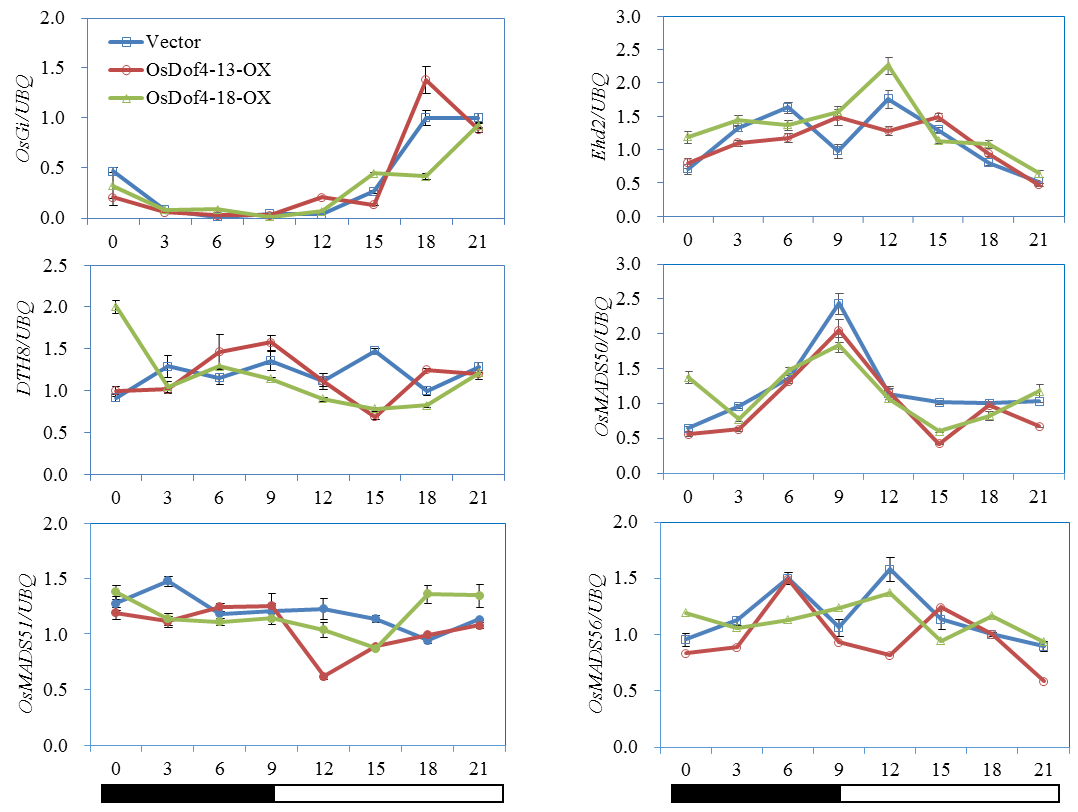

Supplement: Supplementary file 3 — Expression analysis of key floral genes in vector and OsDof4-ox plants under LDs. mRNA levels of flowering genes under LDs were detected by real-time q-PCR. Leaf blades from 35-day-old WT grown under LDs were collected for expression analysis. Blue lines represent for the expression curves of flowering genes in vector plants; Red and green lines respectively represent for the ones in OsDof4-ox-13/−18 plants. Values are means ± SE (n = 3). Error bar indicates SE. (TIFF 2564 kb) [file 12870_2017_1109_MOESM3_ESM.tif]
